# Peptide Targeted by Human Antibodies Associated with HIV Vaccine-Associated Protection Assumes a Dynamic α-Helical Structure

**DOI:** 10.1371/journal.pone.0170530

**Published:** 2017-01-20

**Authors:** Mohammed S. Aiyegbo, Evgeny Shmelkov, Lorenzo Dominguez, Michael Goger, Shibani Battacharya, Allan C. deCamp, Peter B. Gilbert, Phillip W. Berman, Timothy Cardozo

**Affiliations:** 1 Department of Biochemistry and Molecular Pharmacology, New York University School of Medicine, New York, New York, United States of America; 2 The New York Structural Biology Center, New York, New York, United States of America; 3 Vaccine and Infectious Disease Division, Fred Hutchinson Cancer Research Center, Seattle, Washington, United States of America; 4 Department of Biostatistics, University of Washington, Seattle, Washington, United States of America; 5 Department of Biomolecular Engineering, University of California Santa Cruz, Santa Cruz, California, United States of America; Emory University School of Medicine, UNITED STATES

## Abstract

The only evidence of vaccine-induced protection from HIV acquisition in humans was obtained in the RV144 HIV vaccine clinical trial. One immune correlate of risk in RV144 was observed to be higher titers of vaccine-induced antibodies (Abs) reacting with a 23-mer non-glycosylated peptide with the same amino acid sequence as a segment in the second variable (V2) loop of the MN strain of HIV. We used NMR to analyze the dynamic 3D structure of this peptide. Distance restraints between spatially proximate inter-residue protons were calculated from NOE cross peak intensities and used to constrain a thorough search of all possible conformations of the peptide. α–helical folding was strongly preferred by part of the peptide. A high-throughput structure prediction of this segment in all circulating HIV strains demonstrated that α–helical conformations are preferred by this segment almost universally across all subtypes. Notably, α–helical conformations of this segment of the V2 loop cluster cross-subtype-conserved amino acids on one face of the helix and the variable amino acid positions on the other in a semblance of an amphipathic α–helix. Accordingly, some Abs that protected against HIV in RV144 may have targeted a specific, conserved α–helical peptide epitope in the V2 loop of HIV’s surface envelope glycoprotein.

## Introduction

The 25-year evolution of HIV vaccine strategies has been guided in large part by vaccine failures that disabused incorrect notions. The clinical success of previous subunit vaccines (hepatitis B, tetanus, etc.) demonstrated that immunization with the right protein antigen (subunit) is capable of generating a protective antibody (Ab) response in humans. Notably, although neutralizing antibodies are often associated with protection, several vaccines do not elicit neutralizing Abs, while still offering protection [1]. The inability of the first two HIV subunit vaccines tested in large scale Phase III trials to provide protective antibody responses [2, 3] led to the theory that robust cellular immunity was required to control the establishment of HIV infection. A clinical test of this theory, in the form of cytotoxic T-cell elicitation homologous prime-boost strategies, also failed (STEP study and Phambili) [4, 5]. The success of a heterologous prime-boost strategy that combined elements of these two approaches, the RV144 trial, has provided the only Phase III evidence that vaccination can elicit immune responses that can protect humans against HIV infection [6]. In this study, estimated vaccine efficacy was 60% at 1 year, waning to 31.2% by trial endpoint at 3.5 years [7]. Only these HIV vaccine concepts have ever been tested for Phase III efficacy in human subjects. Phase III blinded, randomized, placebo-controlled clinical trials are widely acknowledged to be the gold standard of evidence in medicine. Thus, it can be reasonably argued that only data from the RV144 trial, the only Phase III HIV vaccine efficacy trial to show a significant reduction in the rate of HIV-1 infection, as compared with placebo, can logically be used to generate medical-evidence-based, human subject protection hypotheses. Conversely, it can also reasonably be argued that the RV144 result, which was restricted to a modest, modified intention-to-treat analysis, is too weak to qualify as medical evidence.

**Fig 1:**
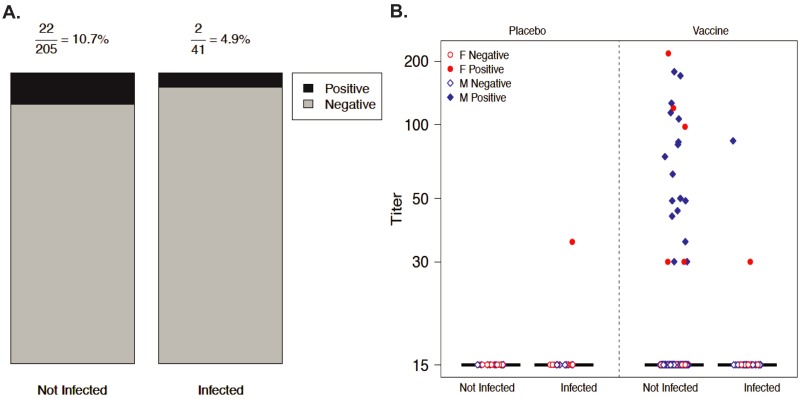
Reactivity of RV144 subject sera with MN peptide. Positive or negative reactivity with the MN peptide, as determined by ELISA titers, for week 26 serum immunoglobulin (Ig) samples from the RV144 case–control study (Haynes et al) with participants stratified according to HIV infection status and treatment assignment. ELISAs were performed as described in Haynes et al. Positive reactivity of a subject’s serum Ig with the MN peptide is defined as a titer (1/serial dilution) of greater than 15, which is the limit of detection for this specific ELISA assay. Values below the limit of detection (LOD) are negative and set to half the LOD. A) Data for vaccinated subjects stratified by HIV infection status. The denominator in the proportion at the top indicates the number of vaccinated subjects (e.g. 205 total subjects included in the RV144 case control study who were vaccinated were not infected with HIV at the end of study follow-up). The numerator in the proportion at the top is the number of subjects whose serum Ig exhibited a positive reaction with the MN peptide. B) Data for both vaccinated and placebo subjects displayed as a dot plot. Sex and immune-response categories are indicated by the color and shape of the points.

The RV144 case-control study was specifically designed to detect correlations between risk of HIV infection and hundreds of vaccine-induced immune factors [6]. Of these factors, three assays detected Abs in the serum of RV144 vaccinees that were targeted at the V2 loop of the HIV envelope protein, gp120, high levels of which were correlated with a lower risk of HIV acquisition. One factor was a primary study variable: an ELISA assay for vaccine serum Abs using the gp70-V1V2^Strain = CaseA2^ protein as the detection probe [6]. The two others were secondary study variables: a gp120 peptide microarray and an ELISA using a non-biotinylated peptide equivalent to the V2 segment from positions 161 to 183 (numbering based on the Hxbc2 reference strain of HIV) of the MN strain of HIV (MN peptide) as the detection reagent [6]. Neither of these two latter probes were glycosylated entities, so they contained only natural amino acids, and they overlapped at the V2 peptide segment from positions 165 to 181[8, 9]. Two independent sieve analysis of RV144 data identified position 169 within this segment as correlating with vaccine efficacy, one of which identified a specific HLA allele/cytotoxic T-cell epitope (CTL) in the MN peptide [10, 11]. Of 205 vaccinees in the case-control study, 24 had Abs reacting with the MN peptide of whom 2 acquired HIV infection, compared to 181 non-reactive with the MN peptide of whom 39 became HIV infected (OR = 0.41). Seen another way, 10.7% of uninfected subjects in the case-control study had high Ab titers against the MN peptide, while only 4.9% of infected subjects demonstrated such titers ([12] and Fig 1A). Intriguingly, if one subjectively chooses to view a vaccinee Ab-MN peptide titer of 30 or higher as a biomarker of protection, out of 24 RV144 vaccinees exhibiting this biomarker in the RV144 trial, 22 were not infected at the time of the RV144 immune correlates analysis (Fig 1B).

Based on these observations, we reasoned that the non-biotinylated, non-glycosylated peptide with an identical sequence to the V2 segment from positions 161 to 183 of the MN strain of HIV contains, within its chemical composition, an epitope targeted by human vaccine-inducible polyclonal Abs that might be capable of protecting against HIV acquisition. Accordingly, we characterized the dynamic 3D structure of this peptide in solution in order to gain insight into the nature of this epitope.

## Materials and Methods

### Sequences

The HIV MN V2 peptide RV144 probe sequence was defined as ITTSIGDKMQKEYALLYKLDIEP, as reported in the RV144 immune correlates study [6]. This peptide was originally developed to demonstrate antibody responses specific to the the MN-rgp120 component of bivalent and multivalent vaccines possessing this protein [13]. V1V2 loop sequence information was obtained from the Los Alamos National Laboratory HIV Database and filtered/selected according to a protocol described elsewhere [14]. Briefly, the initial set of sequences was filtered by removing problematic elements, such as those containing non-amino acid symbols or truncated fragments etc. Finally, a single sequence per patient was randomly selected from the resulting set, while all the others with no reported patient ID, were filtered out in order to minimize bias in modeling the circulating HIV viral population.

### *ab initio* folding

*ab initio* folding was performed using ICM-Pro software (Molsoft LLC, La Jolla, CA, USA) as previously described [9, 15, 16]. Briefly, the 3D atomic structure corresponding to each peptide sequence was built. To optimize the folding simulation, parameters that include the length and temperature (300K) of the simulation, the number of free variables, the minimum gradient and the probability distribution were set to their default values for folding in aqueous solution. The simulation is then performed using a biased probability Monte Carlo method that generates random independent conformations of the peptide. Each conformation is then subject to local minimization. Conformations are ranked based on an energy score derived from energy terms of van der Waals, hydrogen bonding, electrostatics, dihedral angle of deformation, solvation and entropy.

The NMR-constrained peptide folding was performed as described above, with distance restraints among specific atoms obtained from the NMR experiments imposed on the folding simulation. The folding constraints were set to within a range of ± 0.25 Å of these distances (Table 1). The negative control simulation (data not shown) folded the V2 MN sequence in the presence of NMR constraints extracted from an NMR verified ß-hairpin V3 structure [17].

**Table 1:** CYANA NMR-derived atom pairs for which interatomic, inter-residue distances were used as restraints in NMR-constrained peptide folding.

Atom 1	Atom 2	Distance Restraint (Å)
12 Glu HA	13 Tyr HN	3.50
12 Glu HB2	13 Tyr HN	3.82
14 Ala HN	15 Leu HN	3.81
17 Tyr HB1	18 Lys HN	4.23
19 Leu HA	20 Asp HN	2.94
1 Ile HA	2 Thr HN	3.56
21 Ile HB	22 Glu HN	4.22
3 Thr HB	4 Ser HN	4.07
5 Ile HB	6 Gly HN	4.20

### Helicity calculation for folding simulation

Secondary structure was assigned for each conformation in the spectrum of conformations using a modified version of the DSSP algorithm [18] encoded in ICM-Pro. For each folding simulation, the number of helical residues was calculated for each conformation. Helicity was then calculated according to the following formula as the energy-weighted ensemble average of the occurrence of helical residues:

$$H=\;\frac{\sum_{i}\frac{N_{helical\_residues}}{N_{residues}}e^{-\frac{\left(E_{0}-E_{i}\right)}{kT}}}{\sum_{i}\;e^{-\frac{\left(E_{0}-E_{i}\right)}{kT}}}$$

where *k* is the Boltzmann constant, T is the temperature of the simulation (300K), E*i* is the calculated energy of each specific ensemble conformation, E_0_ is the lowest energy conformation in the simulation, H_conf_ is the number of helical residues. This value is therefore zero if no helical residues can be found anywhere in the folding ensemble, which results if the ensemble is populated exclusively by any combination of beta-strand or random coil conformations.

### Homology modeling

The four-stranded β-sheet conformations of V1V2 domain from previously published co-crystal structures of V1V2 with mAbs PG9 and PG16 [19, 20] and the α-helical conformation of V1V2 from the co-crystal structure of V1V2 with mAb CH58 [21] were used as crystallographic templates for the homology modeling of circulating V2 segments. 3D homology models of the V2 variants were built using the ICM Pro software (Molsoft, LLC, La Jolla, CA) according to the following protocol. First, the coordinates of backbone atoms of a V2 peptide were assigned to be equal to the corresponding backbone coordinates of a V2 template. Then, the V2 peptide was subjected to Biased-Probability Monte Carlo (BPMC) sampling of ICM-Pro to produce the lowest energy structure matching the template coordinates. Importantly, the backbone of the model was tethered to the template backbone during the BPMC sampling by imposing additional energy penalty for any backbone atoms deviations. Finally, the energy of the resulting V2 homology model was recorded. Terms for van der Waals, hydrogen bonding, electrostatics, dihedral angle deformation, entropy, and solvation were included in energy calculation.

### NMR sample preparation

The V2 peptide was commercially synthesized at >95% purity by Genemed Synthesis Inc. (San Antonio, TX, USA). The NMR sample was prepared by diluting a concentrated 2.3 mM stock solution of the peptide prepared by dissolving 1 mg of lyophilized powder in distilled water. The final concentration of the peptide employed in NMR data collection was ~230 μM at pH 7.0 in 90% H_2_O / 10% D_2_O.

### NMR data acquisition

All spectra were acquired at 298K on a Bruker *AVANCE II* spectrometer operating at B_0_ field value of 900.26 MHz equipped with a Z-axis gradient TCI CryoProbe. A standard suite of homonuclear two dimensional experiments [22], DQF-COSY, NOESY (mixing time 200 ms) and TOCSY (mixing time 70 ms) were acquired for chemical shift assignments. To assign the aliphatic and aromatic carbon chemical shifts, natural abundance ^13^C-edited 2D HSQC was acquired on the same sample.

### NMR data analysis

The raw NMR data was transformed in Topspin 2.1 from Bruker Biospin and the processed spectra analyzed in CARA 1.5 [23]. The spin-systems were identified by assigning resonances originating from scalar coupled atoms in DQF-COSY and TOCSY respectively. The sequential assignments were confirmed from H^N^(i)-H^N^(i±1) and H^N^(i)-H^α^(i-1) connectivity in the NOESY spectra to aid complete backbone and partial side-chain assignments of the peptide. Dihedral angles were obtained by analyzing H^α^/C^α^ and C^β^ chemical shifts of the V2 peptide in TALOS [24]. Distance restraints between spatially proximate protons (<6 Å) were calculated from the NOE cross-peak intensities using the built-in calibration module from CYANA 2.1 software [25].

## Results

### NMR structural analysis of HIV MN V2 peptide

**Fig 2:**
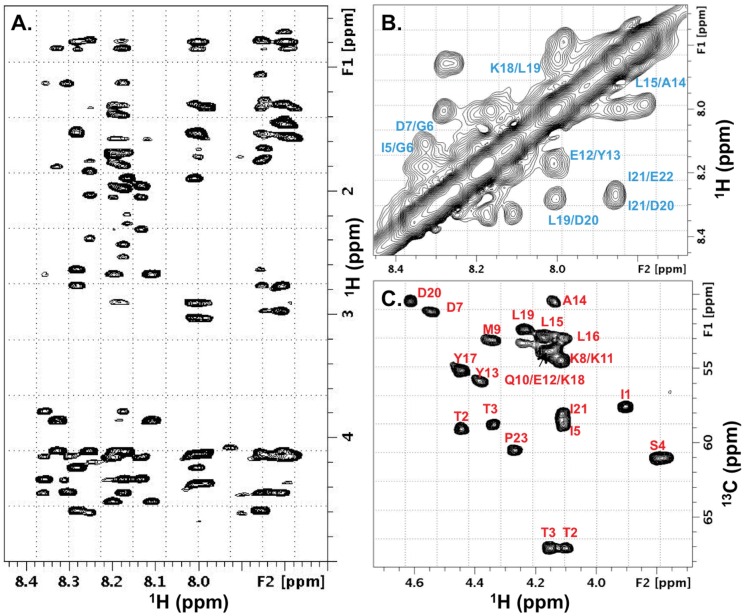
NMR structural analysis of HIV V2 MN peptide supports a propensity for alpha-helical structure. (A) Representative amide proton to side-chain correlations from a 2D homonuclear NOESY spectrum, (B) Inter-residue H^N^-H^N^ correlations from 2D NOESY, and (C) 2D natural abundance C^13^-edited HSQC spectrum of the Calpha region. The residue specific annotations are displayed in panels (B) and (C).

A dilute sample of the non-biotinylated MN peptide in water was used for resonance assignments to minimize the weak propensity for concentration dependent self association observed at higher concentration. The amino acid spin-systems were identified using scalar connectivities and the sequential assignments confirmed in the 2D NOESY spectra to obtain nearly complete backbone (H^N^, Hα, Cα) and partial side-chain resonance information (Fig 2A, 2B and 2C).

### Structure determination from NMR resonances of HIV MN V2 peptide shows α–helix

It is well established that C_α_ and C_β_ resonance positions, which we obtained for the MN peptide, are sensitive reporters of secondary structure. Empirical (matching to the structural database) and specific (predict only highly rigid, canonical secondary structures) standard approaches to determining secondary and tertiary structure from these resonances did not predict obvious secondary structure for the peptide (S1 Fig). However, these approaches would be inconclusive for even slightly distorted or flexible α-helices.

In order to achieve a more dynamic, database-independent picture of the structure of the MN peptide, we utilized an *ab initio* peptide folding algorithm that has previously been used for NMR structure determination and was demonstrated to be highly accurate [15]. Specifically, this algorithm predicted the structure of a 23-residue folded protein domain solely from its sequence, among other successful peptide structure predictions [14–16, 26–28]. This performance proves that the algorithm searches the entire conformational space of any non-aggregating 23-mer peptide dissolved in aqueous solution and has an energy function of sufficient accuracy to identify the native conformation. Furthermore, as all conformations searched are recorded and ranked, a picture of the dynamic ensemble of a non-structured peptide can also be achieved by this method by observing the spectrum of low energy conformations adopted by the peptide.

**Fig 3:**
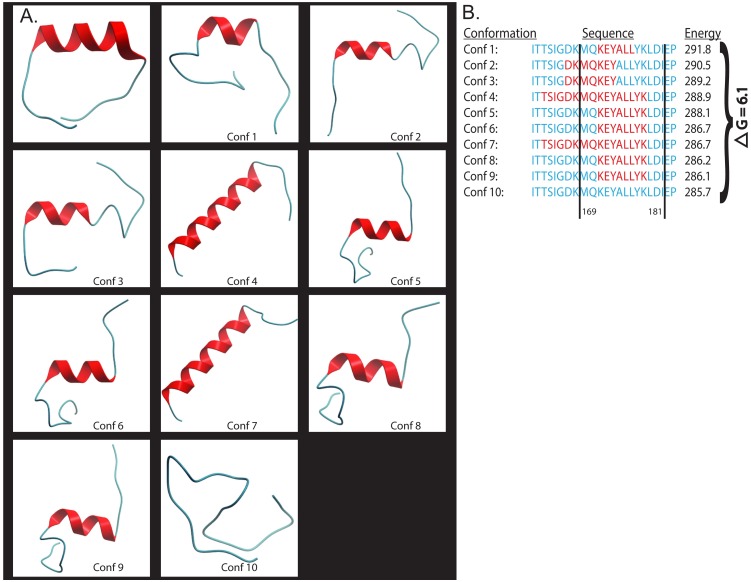
Computational folding of HIV V2 MN peptide shows a preference for the alpha-helical secondary structure. (A). Shows the lowest energy conformation of the *ab*-initio folding (top left) and the top ten conformations of the NMR-restrained ICM folding. Segments of the peptide that adopt alpha-helical structure are depicted in red. (B). Sequence alignment of the top ten NMR-restrained folding conformations with their corresponding energy scores. Red color indicates segments of the peptide that have adopted an α–helical conformation.

We used the inter-residue distance restraints derived from the observed NOE cross-peaks (see Methods) to constrain the *ab initio* folding of the MN peptide. If the results for this constrained folding are consistent with those of the free *ab initio* folding, then the peptide very likely adopts a flexible, distorted α-helical conformation in solution. If, on the other hand, low energy and α-helical conformations cannot be achieved, this would imply the structural features of the peptide in solution as observed by NMR are irreconcilable with the intrinsic biophysical properties of the peptide sequence. This is an extremely stringent test, as even a single, inter-residue, inter-atomic NMR-derived distance that is incompatible with an α-helix will result in a dramatic divergence between the free *ab initio* folding and the NMR-restrained *ab initio* folding due to the strict geometry of peptide inter-atomic interactions. Our results showed that the NMR constrained folding resulted in a spectrum of conformations dominated at the lowest energies by partly α-helical conformations (Fig 3A). The first conformation that did not contain any α-helical turns was 6.1 kcal-equivalent-energy units below the lowest energy conformation, suggesting that the peptide has strong α-helical propensity in its central region, corresponding approximately to the “C” β–strand seen in the crystal structures of the V1V2 domain, with dynamic unfolding at its N- and C- terminal ends (Fig 3B). *ab initio* folding of the MN peptide unrestrained by NMR data also resulted in a spectrum of conformations dominated at the lowest energies by partly α-helical conformations (Fig 3A). A control folding using NMR restraints derived from a V3 loop ß-hairpin structure resulted in unfolding of the α-helix (data not shown). Thus, the NMR restrained and free 3D secondary structural propensity of the MN peptide are convergent and consistent and likely represent the true 3D structure of the MN peptide under ELISA conditions.

### Conservation of the α–helical structure in circulating HIV strains

**Fig 4:**
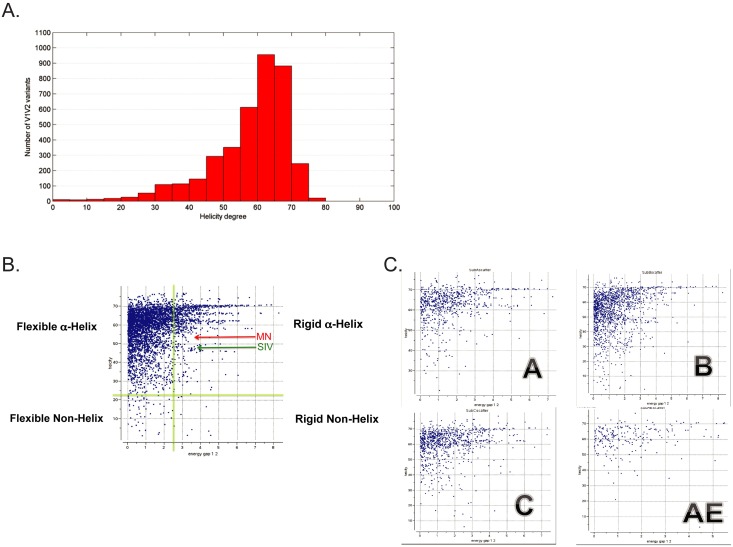
Distribution of the helicity degree of the folded V2^155-176^ variants. 3D structure of each unique V2^155-176^ sequence recorded in the LANL HIV database was predicted using the *ab initio* folding protocol as described in the Methods section, and the helicity degree (in %) was calculated. The higher the helicity degree is, the more residues of a given sequence tend to accept an α-helical conformation.

As our results showed that *ab initio* folding in the absence of NMR data can accurately assess the 3D structure of this V2 segment in a manner that is consistent with experimental NMR observation, we sought to determine if MN’s α-helical conformation is an anomaly or the rule across circulating strains. *ab initio* folding can rapidly determine the dynamic structure of the equivalent V2 segment to that of the MN peptide in all circulating strains. Thus, we folded only the V2 segment from positions 165 to 181 in circulating strains, which was the minimal segment showing an association with risk of HIV infection in RV144 by peptide microarray and also contained in the MN peptide [6, 12]. Folding a larger segment is not justified since each additional amino acid increases the simulation time for these thousands of sequences exponentially, and there is no real gain to the larger peptides based on the hypothesis that both the the MN peptide and the V2 microarray contain the Ab-targeted epitope of interest. The α-helical occurrences, or “helicity”, of the dynamic folding ensemble was calculated for each strain (Fig 4A) along with the energy gap between the lowest energy conformation and the next lowest energy conformation, which is a measure of the flexibility of the variable loop peptide structure in solution (larger gaps, less flexibility) [16, 28]. No representative of any circulating strain showed an absence of helicity, and the vast majority of V2 variants of the peptide from this region strongly preferred an α-helical conformation (Fig 4B). Notably, although this segment from the MN strain was predicted to be relatively inflexible (rigid), the vast majority of the equivalent segments from circulating strains prefer flexible α-helical conformations. This distribution did not change between the four major HIV subtypes (Fig 4C). Interestingly, the equivalent segment from the SIVmac251 strain, which is frequently used in preclinical animal model studies of HIV infection, models closely with the MN strain as a rigid α-helix (Fig 4B). However, the segment models as a flexible α-helix in some of the other SIV strains used in these animal model studies (S2 Fig).

**Fig 5:**
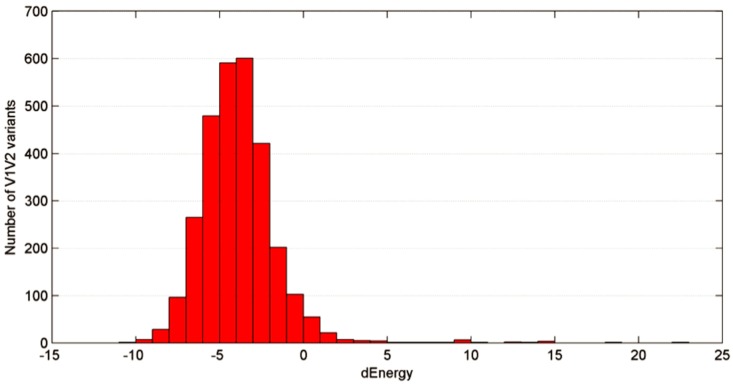
Distribution of the energy difference between the α-helical and β-sheet V2^167-179^ conformations on the informatics of all V2^167-179^ variants. For each unique V2^167-179^ sequence recorded in the LANL HIV database two homology models were built as described in the Methods section, and the difference between the energies of these two models (dEnergy) was calculated by subtracting the energy of the β-sheet model from the energy of the α-helical model. Thus, negative dEnergy scores suggest that the α-helical conformation is energetically favorable in comparison to the β-sheet conformation.

To further confirm this strong preference for an α-helical conformation locally at V2^165-181^, we modeled the set of circulating V2 variants of this segment onto the crystallographic structures of this segment, which appear as a ß-strand in complex with Abs that broadly neutralize HIV viruses *in vitro* and as an α-helix in complex with an Ab that does not significantly neutralize HIV viruses *in vitro*. Specifically, for each unique V1V2 sequence reported in LANL we constructed two homology models: one based on the α-helical conformation observed in complex with mAb CH58 [21], and the other based on the β-sheet fold, observed in complex with mAb PG9 [19]. Only the V2 region crystallographically resolved in both crystal structures (positions 167 to 179 of HxB2 numbering) was modeled. A total of 5810 homology models were built, and the distribution of energy scores between α-helical and β-sheet models was compared (Fig 5). The resulting data suggest that the α-helical fold of V2^167-179^ is energetically favorable for the majority (>95%) of the modeled V1V2 variants, in concordance with the high-throughput folding. We also did not detect any substantial difference in V2^167-179^ structural preference among different most prevalent HIV subtypes and CRFs by this method (Table 2). Accordingly, by two independent methods, the local conformational preference of the V2 segment likely targeted by anti-HIV Abs is α-helical.

**Table 2:** Quantification of the structural preference of the α-helical V2^167-179^ conformation for the β-sheet conformation.

HIV Clade	Median dEnergy
Subtype A	-5.40
Subtype B	-3.50
Subtype C	-3.90
Subtype D	-3.75
Subtype G	-4.05
CRF01_AE	-3.70
CRF02_AG	-4.50
All clades	-4.00

### Sequence conservation pattern in the context of α-helical structure

**Fig 6:**
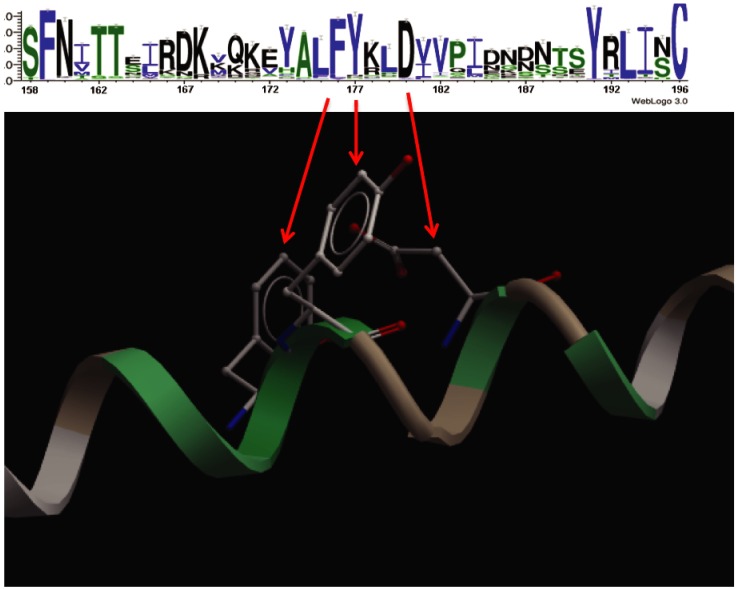
Sequence conservation in the context of alpha-helical structure. The sequence logo of the V2 loop of circulating HIV strains showing positional conservation of residues in the loop. The most highly conserved residues in the V2 loop map to the same face of the alpha-helical structure in 3D space as shown by the red arrows.

In this region of the V2 loop, amino acid positions at which many substitutions take place across circulating HIV strains alternate irregularly with positions in which the consensus amino acid is fairly invariant. Mapping this pattern of positional conservation and variation onto a canonical α-helical scaffold reveals that the conserved positions cluster in 3D space to one specific surface of the α-helix (Fig 6).

## Discussion

We investigated the three-dimensional structure of a 23-mer, non-glycosylated peptide that appears to react specifically with Abs hypothesized to protect human subjects from HIV acquisition. NMR analysis of the peptide shows that the peptide is dynamic and does not adopt a rigid, canonical secondary structure. Integration of the NMR data with *ab initio* folding simulations, and structural and informatics analyses, however, suggests that this peptide preferentially adopts a dynamic, distorted α–helical conformation in solution corresponding to approximately positions 163 to 178 of the V2 loop. Accordingly, this structural analysis could not have been achieved by any of the more common structure determination methods alone (NMR, crystallography, cryo-electron microscopy).

Our studies suggest that the counterparts of this peptide in the V2 loops of circulating HIV strains also consistently prefer α–helical conformations that vary in flexibility. This finding provides a structural basis for hypothesizing that the epitope targeted by the MN-specific, RV144-vaccine-elicited, protection-associated Abs is conserved across circulating HIV strains. Indeed, independent evidence suggests that some protective Abs observed in the RV144 trial react with an Ab-targeted epitope that is conserved across multiple HIV subtypes[8, 29].

Part of the V2 segment represented by the MN peptide was already reported to be structurally polymorphic. It appears as a β–strand conformation in crystallographic structures of the V1V2 domains from several strains and in the crystallographic structure of the gp120 trimer from the HIV strain BG505. These crystallographic structures were all produced from complexes of these HIV domains with neutralizing Abs. Alternatively, part of this segment appears as an α-helix in complex with the RV144 vaccinee derived mAb, CH58 [19, 21, 30]. CH58 does not react *in vitro* with the MN peptide we studied here nor with multiple HIV subtypes, including the CaseA2 strain, and neither does the closely related mAb CH59 [21]. Thus, the conformations of this V2 segment, as exhibited in complex with CH58/59, although they are α–helical, are not precisely representative of the MN peptide epitope targeted by putative, protective anti-HIV Abs. Interestingly, CH58 and CH59 are poorly or non-neutralizing. A previous study strongly correlated β–strand propensity in the V2 loop with neutralization [31], but protection in RV144 correlated with TZM.bl-measured, viral neutralization *in vitro* only in the presence of low plasma, gp120-specific IgA [6]. Our study potentially correlates the α-helical conformation with non-neutralizing functions, including those potentially associated with protection.

There are, nevertheless, several minor uncertainties in our results. Although the peptide used in our studies was identical in composition to that used in the RV144 immune correlates analysis, there is no guarantee that it behaved similarly in the NMR solution as compared to the RV144 analysis ELISA environment. Similarly, flexible peptides are easily induced to different conformations by Abs, so there is no guarantee that the dynamic, distorted α-helical conformation we observe is preserved upon binding of the putative HIV-protective Abs. Finally, our analysis of circulating strains assumes that this segment of the V2 loop is flexible enough to fold as an unrestrained, free peptide, as seen for the V3 loop crown [14, 16, 28], which may not be the case *in situ* in gp120 or in the trimer. Nevertheless, these uncertainties are likely insignificant, especially given the independent incompatibility of these sequences with ß–strand conformations (Fig 5), the independently assessed α-helical propensity across circulating strains (Fig 4) and the compatibility of the positional amino acid conservation pattern across circulating strains in this segment with an α-helix (Fig 6).

Despite their paramount importance to HIV science (and therefore to global health), clues leading to protection-associated, Ab-targeted epitopes are exceedingly rare in the HIV vaccine field despite almost 30 years of intense scientific research. Our results allow us to reliably infer specific molecular details of one protection-associated HIV epitope: it is a non-glycan epitope residing in the V2 loop between positions 165 and 181 and presented on an α–helical scaffold that is conserved across circulating strains. A description to this level of molecular detail of a protection-associated HIV epitope has not previously been reported. The markedly higher frequency of reactivity to gp70-V1V2^Strain = CaseA2^ suggests that other Ab-targeted epitopes are also associated with protection. The implication for other immune correlates, including cell-mediated responses, is that they are composites or aggregates of specific molecular responses like the ones described in this report. Thus, our results may provide new insights for HIV vaccine design.

## Supporting Information

S1 Fig Chemical shift analysis of the V2 peptide in the software TALOS against a database of known high-resolution structures shows a dynamic conformation with no evidence of persistent secondary structure. (A) Predicted Random coil Index (RCI) S^2^ values indicate backbone dynamics with S^2^ = 0 corresponding to mobile and S^2^ = 1 relatively rigid regions. (B) Predicted secondary structure with extended configurations favored in the Ramachandran ϕ/ψ plot indicated in red. The corresponding S2 < 0.7 to be noted as strong indication of a dynamic configuration.
(TIF)

S2 Fig Distribution of the helicity degree of the folded V2^155-176^ segment in SIV strains. 3D structure of each unique SIV V2^155-176^ sequence recorded in the LANL HIV database and the Swiss-prot section of Uniprot was predicted using the *ab initio* folding protocol as described in the Methods section, and the helicity degree (in %) was calculated. The higher the helicity degree is, the more residues of a given sequence tend to accept an α-helical conformation. Some SIV strains commonly used in non-human primate studies are indicated with red dots.
(TIF)
