# Supplementary figures and images for: Peptide Targeted by Human Antibodies Associated with HIV Vaccine-Associated Protection Assumes a Dynamic α-Helical Structure
Source: PLoS One. 2017 Jan 20;12(1):e0170530. doi: 10.1371/journal.pone.0170530 (PMC5249078; doi:10.1371/journal.pone.0170530)

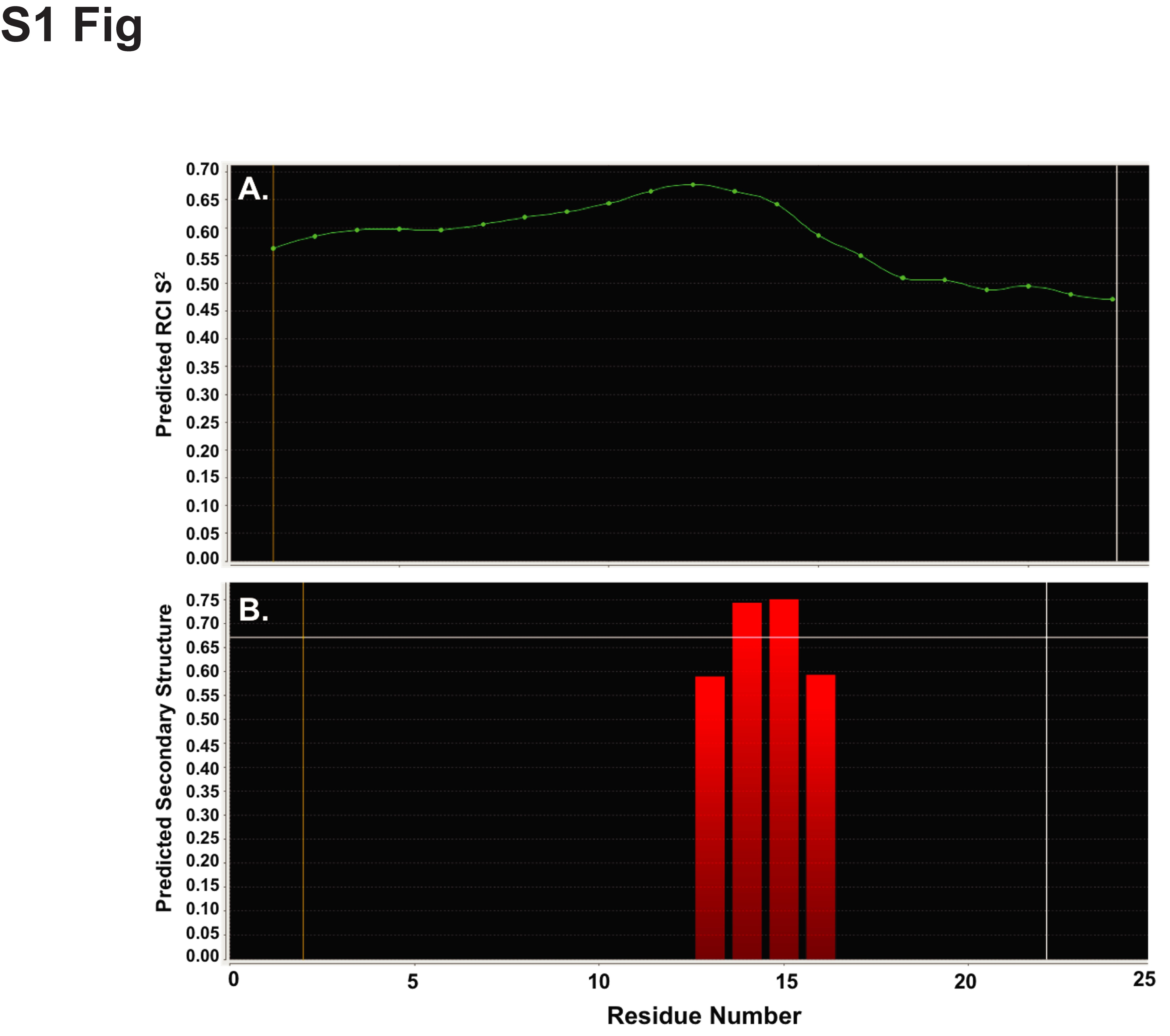

Supplement: S1 Fig — (A) Predicted Random coil Index (RCI) S2 values indicate backbone dynamics with S2 = 0 corresponding to mobile and S2 = 1 relatively rigid regions. (B) Predicted secondary structure with extended configurations favored in the Ramachandran ϕ/ψ plot indicated in red. The corresponding S2 < 0.7 to be noted as strong indication of a dynamic configuration. (TIF) [file pone.0170530.s001.tif]

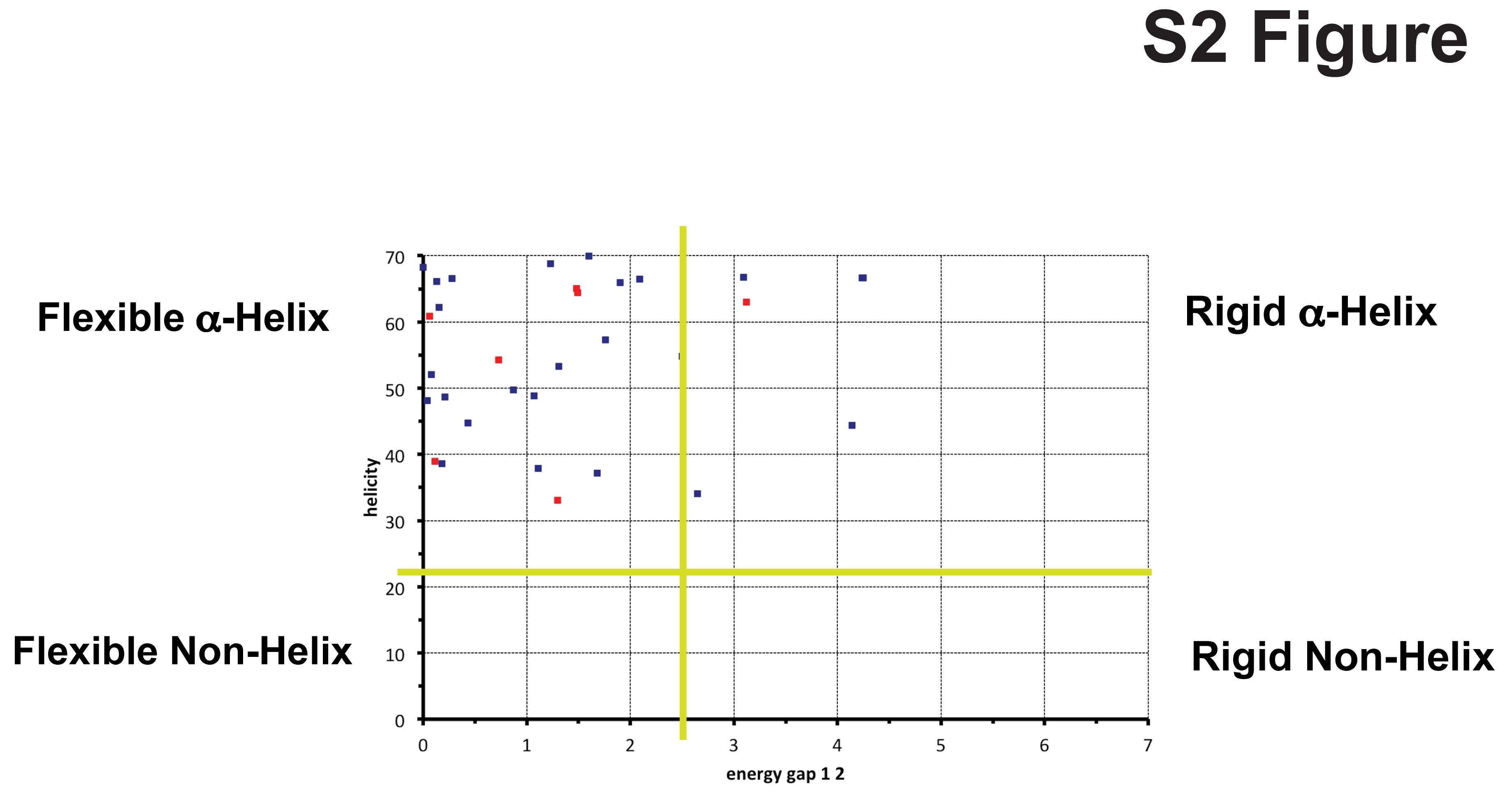

Supplement: S2 Fig — 3D structure of each unique SIV V2155-176 sequence recorded in the LANL HIV database and the Swiss-prot section of Uniprot was predicted using the ab initio folding protocol as described in the Methods section, and the helicity degree (in %) was calculated. The higher the helicity degree is, the more residues of a given sequence tend to accept an α-helical conformation. Some SIV strains commonly used in non-human primate studies are indicated with red dots. (TIF) [file pone.0170530.s002.tif]
